# TRialing individualized interventions to prevent functional decline in at-risk older adults (TRIIFL): study protocol for a randomized controlled trial nested in a longitudinal observational study

**DOI:** 10.1186/1745-6215-14-266

**Published:** 2013-08-20

**Authors:** Karen Grimmer, Julie Luker, Kate Beaton, Saravana Kumar, Alan Crockett, Kay Price

**Affiliations:** 1 International Centre for Allied Health Evidence, University of South Australia, GPO Box 2471, Adelaide, South Australia, Australia; 2 Exercise for Health and Human Performance, University of South Australia, Adelaide, South Australia, Australia; 3 Safety and Quality in Health Research Group, University of South Australia, Adelaide, South Australia, Australia

**Keywords:** Functional decline, Aging, Screening, Prevention, Patient-centered care

## Abstract

**Background:**

Functional decline (FD) is a largely preventable feature of aging, characterized as gradual erosion of functional autonomy. This reduces an older person’s capacity for safe, independent community living. The healthcare needs of an unprecedented aging population places pressure on health systems to develop innovative approaches to ensuring older people live healthy and independent lives for as long as possible.

TRIIFL aims to demonstrate that:

1. Incipient FD in older people can be identified using a simple telephone-screening process within four weeks of discharge from an emergency department presentation for a minor health event; and

2. Early engagement into a person-centered individualized intervention arrests or reduces the rate of FD over the next 12 months.

**Methods/Design:**

A randomized controlled trial (RCT) nested within a 13-month longitudinal cohort study. The RCT (conducted over 12 months) tests the effectiveness of a novel, early, home-based, personalized program (compared with no intervention) in arresting or slowing FD.

TRIIFL focuses on older adults living independently in the community, who have not yet had a serious health event, yet are potentially on the cusp of FD. Participants in the longitudinal cohort study will be recruited as they present to one large tertiary hospital Emergency Department, providing they are not subsequently admitted to a ward. Sample size calculations indicate that 570 participants need to be recruited into the longitudinal study, with 100 participants randomized into the trial arms.

Measures from all subjects will be taken face-to-face at baseline (recruitment), then subsequently by telephone at one, four, seven and thirteen months later. Measures include functional abilities, quality of life, recent falls, mobility dependence, community supports and health service usage. Specific to the nested RCT, the quality of life tool (SF12) applied at one month, will identify individuals with low mental component quality of life scores, who will be invited to enter the RCT.

Assessors will be blinded to RCT arm allocation, and subjects in the RCT will be blinded to the intervention being received by other subjects.

**Trials Registration:**

Australian & New Zealand Clinical Trials Registry: ACTRN12613000234718

## Study aim

1. To determine whether an individualized early intervention for community-dwelling older people with poor mental component quality of life scores measured one month after discharge directly from a hospital emergency department (ED), reduces their likelihood and/or rate, of functional decline (FD) over the next twelve months.

2. To demonstrate that:

a. Incipient FD in an older person can be identified within four weeks of discharge from an ED presentation for a minor health event, using lower than median mental component quality of life scores; and

a. Engaging these older people early, into a person-centered home-based intervention, arrests or reduces the rate of FD over the next 12 months.

## Background

The immediate future presents Australian and global health policy challenges in how to meet the health needs of an aging population of unprecedented size [1]. Prevention rather than treatment is a critical requirement. The potential for FD with increasing age is outlined by Edwards *et al*. [2], who illustrated that with age, an increasing number of people are classified as below the ‘disability threshold’, putting them at significant risk of preventable health crises such as falls, malnutrition and/or infections. In 1950, less than 1% of the global population was over 80 years old, but by 2050 this proportion is expected to be 4%. The greatest increase will be in Organisation for Economic Co-operation and Development (OECD) countries where the portion of the population over 80 years old is expected to increase from an average 4% to 9.4% [1]. In Australia the number of people 85 years + will quadruple from 0.4 million in 2010 to 1.8 million in 2050 [3]. In 2009 to 2010, Australian government spending on aged care was around $11 billion; two thirds of that was in residential aged care [4]. Institutional care use and acute care support needs of older people will place commensurate pressure on health systems to develop innovative new approaches to ensuring older people live healthy and independent lives for as long as possible [1].

### Functional decline

FD is a largely preventable feature of aging, characterized as gradual erosion of functional autonomy, which reduces an older person’s capacity to live safely and independently in the community [5-7]. FD encompasses both physical and cognitive function. It is commonly accepted that FD precedes frailty [8]; however, there is no standard approach for defining or measuring either state [8,9]. Reduced functional ability is associated with increased mortality and morbidity rates, increased use of health services in all sectors and greater rates of institutionalization [7,10,11].

There are a number of Australian federal and state health and aged care service initiatives to support ‘aging in place’ , aiming to optimize older people’s health, dignity, confidence, self-esteem and independence. However, these initiatives largely center on older people ‘known to the system’ [12]. Many older people on the cusp of FD do not come to the attention of healthcare providers until they are in health and/or social crisis [2], and by then it may be too late for effective intervention. Even individuals who are already receiving community services at home, such as Government-funded community care packages, may not be receiving the care required to prevent FD [13].

The current lack of focus on early FD results from: a) the lack of comprehensive primary health capture points where older people are routinely screened for functional and cognitive capacity; b) no comprehensive, timely or sensitive screening tool to identify older people with incipient FD; and c) no early-intervention program which has been shown to have long term effectiveness to prevent, or slow, the rate of, FD.

### The importance of detecting FD after an ED presentation

Evaluation of recent changes to the way that general medical practitioner (GP) services are being provided in Australia (for example, by large corporate health services or in multidisciplinary community clinics) suggest that many older people now do not have regular contact with the same GP [14,15]. This potentially attenuates opportunities for regular monitoring of older persons’ functional abilities or flagging the need for intervention before FD becomes critical. In Australia, decreasing opportunities and incentives to maintain contact with a regular GP may be related to the increasing number of frail, community-dwelling older people who appear to rely on hospital EDs for crisis health management [16]. These individuals often do not require admission to hospital for acute care. Rather, their needs are more for health assessment and short-term management strategies and for linkages to appropriate community supports to assist them to maintain safe community independence in the longer term. Thus, whether or not it is the right place, ED attendance without a subsequent hospital admission, is an increasingly important service-contact point related to incipient FD.

### How is FD detected?

FD generally occurs subtly and can remain undetected until an unexpected and often catastrophic event occurs [6]. This can be a fall or unexplained ill health which requires hospitalization. This event, if followed by a comprehensive assessment, can expose the magnitude of an individual’s loss of capacity to function safely and independently [6,7,17].

There are a variety of ways of assessing FD; however, it is most commonly assessed as the capacity to complete basic and instrumental activities of daily living (ADLs and iADLs). Basic ADLs are everyday tasks (bathing, dressing, feeding, continence, transferring, toileting) [18], while iADLs are higher level activities (shopping, driving, banking and so on) [19,20]. Inability to manage ADLs safely and independently may only be temporary, if related to illness. This will usually respond well to recovery time and short-term targeted intervention. FD, however, may be irreversible if identified too late for effective community-based interventions. Individuals who have passed the critical threshold of FD may not be able to live safely at home despite intensive and long-term supports and, thus, may require permanent residential care. Irrespective of the point at which FD is assessed on its trajectory, there is no agreement on the best measures to detect FD nor the point of critical deterioration [21].

Detecting FD early and putting supports in place, as directed by the older person, to redress specific and individual areas of decline has been proposed as a way of maintaining older people’s community independence for a longer time [17,22-25]. Comprehensive frailty indices with multiple screening items have also been proposed from population research, mainly conducted in the UK and Canada [6,21,26,27]. However, there are few opportunities in Australia comprehensively to capture this amount of information from community-dwelling older people who are not in health crisis and who may not routinely come to the attention of the healthcare system. FD screening tools reported in the Australian literature are implemented at ‘point-in-time’, commonly during a health crisis at an ED presentation or hospitalization [28]. In these environments, assessment of FD may be inappropriate, when older people are unwell, disoriented, frightened and/or in unfamiliar environments. This raises issues of validity of assessment and/or reliability of responses.

### Pilot work underpinning this protocol

The TRIIFL study builds on 15 years of research into improving the quality of discharge planning for older people, to ensure that older people were transferred safely from ill health in a hospital bed to health at home [25,28-36]. Our work has highlighted common barriers of lack of ownership and collective responsibility for the problem, lack of time, poor communication and resources, and hospital/community ‘turf wars’. Sadly there have been no real changes in discharge planning standards or outcomes in Australia over this time.

We recently published findings from an innovative longitudinal pilot study we conducted in 2011, into prediction and downstream assessment of FD in older people presenting to, and being discharged from, a large South Australian tertiary hospital ED [37]. This study used a comprehensive data collection process to recruit individuals who were not admitted to a hospital ward, that is, they were discharged directly from ED usually within four to eight hours of presentation. This work clearly showed that ED was the choice for 30 to 60 older people/day, who were eligible for our study and did not have their own GP. In our pilot study, we followed up subjects at one week and one and three months later to follow the course of FD. Estimated likelihood of FD at discharge was made with a comprehensive purpose-built assessment tool, as well as the Hospitals Admission Risk Profile (HARP) instrument [36]. We reported that overall, the likelihood of FD at discharge approximated 50%, significantly more than the 30% predicted in the Australian Discharge of Elderly from the Emergency Department (DEED) study [38]. Of note in the 2011 pilot study was the percentage of ‘younger’ old patients (65 to 75 years old) who had significant risk of FD, despite attracting no age weighting from the HARP instrument.

The TRIIFL protocol is based on a novel finding from this pilot research, that subjects with lower than median mental component scores (MCS) on the Short Form-12 Health Survey (SF12) quality of life instrument [39,40], administered one month post-discharge from the ED, were significantly more likely to demonstrate FD two months later (identified as significant reduction in iADL scores, increased falls and hospitalizations), compared with individuals with above-median MCS scores at the same time point. We propose that if early intervention was offered to individuals with low mental health quality of life scores at this point in time, progression of FD could be altered.

## Summary

Identifying and effectively reducing FD in community-dwelling older Australians is of significant interest to policy makers and clinicians. To date, there is no valid or reliable way of identifying FD in any setting, and FD identification usually occurs at crisis health presentations to hospital, rather than in community health settings under ‘usual’ circumstances. Thus, many individuals go undetected, until their capacity to live independently and safely in the community is in question. Our research is the first that we know of, to propose that a real risk of downstream FD in an older person can be identified within four weeks of an ED presentation for a minor health event, using low mental quality of life scores. We further propose that early community-based interventions for people identified as having risks of FD can prevent or delay its onset.

### Current status

A competitive national grant application has been made to support project funding for two years from 2014.

## Methods

### Study design

**Figure 1:**
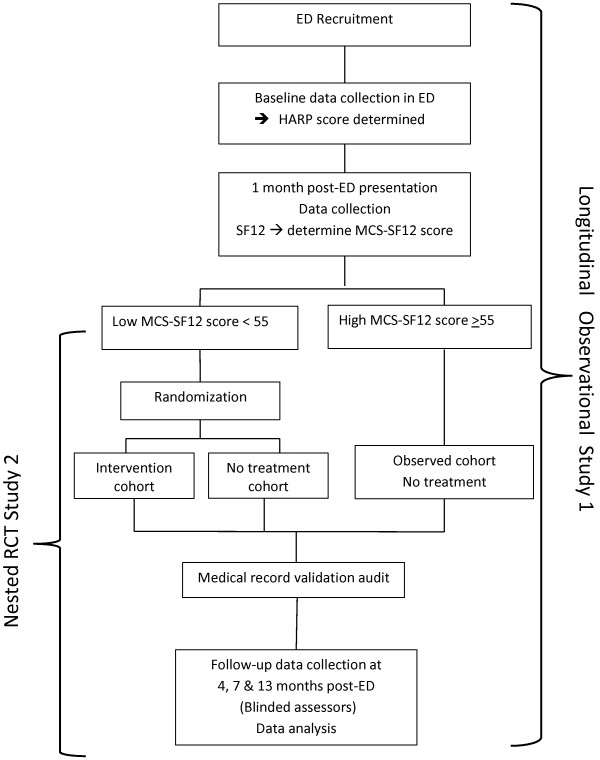
Study design – RCT nested in a longitudinal cohort study.

TRIIFL is a randomized controlled trial (RCT) nested in a 13-month longitudinal cohort study (Figure 1).

#### Study 1: Longitudinal cohort study

The total sample is recruited in Study 1. Every participant will be assessed for FD outcomes and predictors over the following 13 months (at five time points) using a battery of tests.

### Setting, timing

Eligible individuals will be recruited from the ED of a large metropolitan hospital in Adelaide, South Australia, between April and June 2014, using the recruitment process successfully piloted in our 2011 study [37].

### Eligibility criteria

Inclusions

● 65 years old or older

● Presented to ED with non-catastrophic health conditions which do not result in admission to hospital for further care

Exclusions

● Suffering communicable diseases requiring isolation

● Current mental health crisis

● Under detention

● Diagnosis of dementia

● Unable to communicate in English

● Profoundly deaf (such as would limit telephone communication at follow-up)

To remain eligible for the study, individuals must not subsequently become an inpatient as a result of the index ED presentation (that is, must be discharged directly to home from ED). Subsequent hospital admissions and/or ED presentations (unrelated to the index ED presentation) do not compromise eligibility, as this information, captured at each follow-up, is important to build a service-usage profile. Being a ‘frequent flyer’ to ED does not compromise eligibility, as these individuals may well be on the cusp of FD and are not detected, despite multiple ED presentations.

### Baseline screening

We will collect data in the ED (baseline) on consenting, eligible participants from

•the electronic hospital admissions system - age, gender, residential details, differential diagnosis; and

•a test battery as successfully piloted in our 2011 study [37]. This captures the highest level of schooling, primary language, ED presentations and hospitalizations in the past six months, falls in the past twelve months using the Falls Risk in Older People - Community (FROP_COM) instrument [39], living arrangements, requiring a carer, receiving community services, type and use of gait aid, iADLs[19] and cognition with the Mini Mental State Examination (MMSE) [40].

Based on data from our pilot study, we will additionally apply the Katz *et al*. [18] 20 basic ADL instrument and two quality of life measures – the SF12 [41,42] and the Australian Quality of Life (AQoL 4D) [43]. By collecting both basic and iADL scores, we can provide comprehensive ADL information about the target population to compare with international data. The SF12 provides the opportunity for international population-based comparisons of quality of life [41,42] with our sample of older ED presenters. The AQoL, which has not previously been used for older people discharged from ED, provides an Australian perspective on health-related multi-attribute utility quality of life [43], and allows production of health cost effectiveness data (quality-adjusted life years (QALYs)), and estimation of burden of disease.

This total test battery takes an average of 5.5 minutes to administer. All but two questions are delivered verbally by the recruiting researcher, who records the answers. The older person only has to write responses to two MMSE questions.

### Outcome measures

At one, four, seven and thirteen months after recruitment, all participants will be contacted by telephone and consent sought for interview. Socio-demographic information will be confirmed, and all outcome instruments and mutable independent variables will be recorded (Table 1). Semi-structured questions will be asked about how participants are managing, their concerns and reasons for subsequent hospitalizations and ED presentations. At the four, seven and thirteen month data collection points (after nested RCT commencement), assessors will be blinded to group allocation.

**Table 1:** Timeframes of outcome measurements

**Measures**	**Baseline**	**1 month**	**4 months**	**7 months**	**13 months**
ADLs	√	√	√	√	√
iADLs	√	√	√	√	√
SF12	√	√	√	√	√
AQoL_4D	√	√	√	√	√
Falls	√ (last 12 months)	√ (last month)	√ (last 3 months)	√ (last 3 months)	√ (last 6 months)
Hospitalizations	√ (last 6 months)	√ (last month)	√ (last 3 months)	√ (last 3 months)	√ (last 6 months)
Gait aid (type)	√	√	√	√	√
Living arrangements	√	√	√	√	√
Carer engagement	√	√	√	√	√
Organized (formal) community services	√	√	√	√	√
GP visits	√	√	√	√	√
Informal community supports	√	√	√	√	√
Satisfaction with community supports	√	√	√	√	√

ADLs, activities of daily living; AQoL_4D, Australian Quality of Life; GP, general practitioner; iADLs, instrumental activities of daily living; SF12, Short Form-12 Health Survey; √ indicates an occasion of data collection.

### Additional data and calculations

From baseline information on age, iADLs and MMSE scores, a HARP score will be determined [17,36]. HARP, while sensitive to approximately 63% only, is useful for comparing FD risk in our sample with other Australian and international studies. Validation details of the index ED presentation will be undertaken via patient record audit to verify age, presenting diagnosis, previous hospitalizations and ED discharge plans (if any).

### Study 1 analysis

FD will be assessed as change from baseline at each time point as:

•One or more falls increase from the baseline rate

•More than one event of hospitalization, or ED presentation, from the baseline rate

•Decrease of one point in any item of the ADL instrument [18], or

•Decrease of 2+ points from baseline total iADL score [19], or any decrease in baseline score in the domains of home activities, doing laundry, shopping and getting places (we previously reported these domains as critical to functional independence) [37]

•Scores decreasing from above median at baseline in either physical or mental domain, to below median in the SF12 [41,42]

•Any decrease in AQoL_4D (in the manner recently described for chronic obstructive pulmonary disease (COPD))[44]

•Change to a more assistive gait aid (for example, a one point stick to a four-pronged stick)

•Change in living arrangements to more supported care

•Increased carer involvement

•Greater type and frequency of use of formal community services or informal supports

•More frequent GP attendances.

### Study 2: nested RCT

#### Subject selection and randomization

All participants in Study 1 will be stratified at one-month telephone follow-up into low and high scores on the MCS domain of the SF-12, (SF12-MCS), using the median cut point. We previously reported this as a transformed score of 55 [37], which is comparable to the same age-gender population SF-12 norms [41]. The nested RCT will be conducted involving only the subjects with lower than median SF12-MCS scores at this time point (Figure 1).

An independent trials office will randomize these subjects using a computer generated random number series, into an individualized person-centered community-based intervention, or ‘usual care’ which reflects no intervention. It is unlikely that any intervention would normally be offered to this population as it is not current practice to screen people for low mental component quality of life scores after an ED contact.

Considering Studies 1 and 2, there are three study cohorts (Figure 1):

•Subjects with SF12-MCS scores ≥55, who will be observed only, for 13 months after recruitment (12 months after the one month SF12 scores are taken) (the observed cohort)

•Subjects with SF12-MCS scores <55, from which two RCT groups will be allocated one month after recruitment, randomized into

•Intervention specifically tailored to address the individual’s problems, concerns, and goals (low SF12-MCS, intervention cohort)

•No treatment (low SF12-MCS, no treatment cohort).

Outcome measures for the two RCT groups will be taken at three-, six- and twelve-month time points after randomization (four, seven and thirteen months post-recruitment).

### The intervention

The intervention arm applies a novel person-focused home-based approach targeting individual need [45]. Patient/person-centered care has been defined by Berwick [46] as ‘The experience (to the extent the informed, individual patient desires it) of transparency, individualization, recognition, respect, dignity, and choice in all matters, without exception, related to one’s person, circumstances, and relationships in health care’ [46],p2].

The intervention will be applied independently by a large provider of community aged care in South Australia (Elderly Citizens Homes (ECH)). This group has developed a patient-centered program for community-dwelling older individuals considered by their GP to be at-risk of declining functionally. The program involves a home assessment and interview conducted by occupational therapists or physiotherapists to identify individual problems and concerns and to set appropriate goals. Care options are discussed with older people and families in particular individualized interventions, which could include attendance at day therapy centers, exercise, fitness, balance-retraining and/or socialization, organization of home help or community care packages, motivational interviewing or counseling. Transport to programs can also be provided to increase compliance and reduce attendance barriers. Programs can last three to fourteen weeks, depending on need. The median/person cost of the intervention approximates $2,100 (interquartile range (IQR) $350), which approximates the cost of one acute South Australian hospital bed-day.

### Ethical issues

Written permission has been obtained from the Hospital Executive and ED Management of the research site, and ethical approval has been granted by the Human Research Ethics Committee of the University of South Australia (Application ID: 0000031475).

While there are indications from its early evaluation data that the ECH intervention is acceptable to clients, its effectiveness is largely unproven, particularly in individuals with low SF12-MCS scores at commencement. We do not know that the intervention will provide any advantage; in fact, individuals may perceive that they are subjected to additional burdens, such as unwanted assessments and visitors in their home for (perhaps) no overall benefit. We see no disadvantage for the ‘control’ group (low SF12-MCS, no treatment cohort) at this point in our understanding, in not being offered care, nor not knowing that the intervention is available, as they are currently not offered routine intervention so early after an ED discharge. If ,however, at subsequent follow-up points (three, six or twelve months), any participant in the observed cohort arm or the low SF12-MCS, no treatment arm is identified as potentially demonstrating FD on any study measure, the need for rescue care will be suggested and ways to access this discussed. This may include attendance at their GP or making contact with local community service providers.

### Sample size considerations

To date, there is scant research that has established the effectiveness of any community-based person-centered intervention program that alerts older people who are on the cusp of FD to impending FD and provides means to assist and support them. We drew the likely effectiveness of the proposed intervention program from research on early intervention programs for at-risk older people who are in hospital or rehabilitation units. This included an Australian study [47] (significant improvement in goals and functioning) and a Dutch study [48] (effect size 0.25). We acknowledge that this research may not be readily generalizable to estimate effectiveness of the intervention in our reference population; however, it is the best available evidence for sample size calculation.

Our nested RCT study sample size was further calculated on minimal differences in iADLs and SF12 scores, expected at three-month follow-up from our pilot data [37]. These estimates are based on the three-month follow-up distributions for iADLs and SF-12. Parameters of power were set at π = 80% (1-β), with α = 5%. For iADLs, we identified that a minimum absolute effect size of 1.2 would be needed for participants to have dropped by at most 1 point on the Lawton scale (the Lawton definition of FD requires a drop of 2+ points on the scale). Thus, a minimum sample size of N_IADL_ = 50 per RCT cohort would be required (total 100). For the MCS domain of SF-12, a minimum absolute effect size of 4.3 would need to be observed, to put all scores above or equal to the median value, giving sample size of N_MCS_ = 30 per RCT cohort (total 60). Similarly for the PCS domain of SF-12, the minimum absolute effect size of 12.2 gives a sample size of N_PCS_ = 6 per RCT cohort (total 12). We require significance to be determined both clinically and statistically, so over-sampling is not of concern and, therefore, we choose the highest sample size (N_IADL_) of N = 50 per RCT cohort (100 total) at three months RCT follow-up (four months from ED recruitment). Thus, our continuing RCT sample at three months will reflect 100 individuals with low SF12-MCS (randomized 50 each into intervention and control arms), and 100 individuals with high SF12-MCS who will simply be followed-up.

To provide the nested RCT cohort sample, there are specific considerations which will influence the number of individuals invited to participate in Study 1:

•Eligibility at time of recruitment (12% continuation).

•Consent at time of recruitment (30% continuation).

•Continuity through study (43% continuation).

•Study-designed attrition (estimated 10% attrition).

•Increased awareness of the study purpose, through the previous successful relationship established between the research team and the Royal Adelaide Hospital (RAH) staff, regarding awareness of detecting the risk of FD (estimated as improving the capture rate of eligible individuals by 10%).

Thus, to provide a continuing sample of 200 total subjects (with high and low SF12-MCS) at three months follow-up, we require a continuing eligible sample at the one-month assessment point of 220+ individuals; therefore we require a sample of 570 eligible consenting individuals recruited on discharge from ED. Based on previous experience, this will take 14 weeks at the participating hospital.

For the nested RCT, we also took into account an assumed rate of refusal for the intervention of 1:10 individuals who are offered it. This figure is based on pilot feedback from ECH, the intervention provider. We will address a potential loss of power in our intervention arm, which would occur if eligible subjects refused, by replacing every second refuser in the intervention group with an individual randomly selected from the control group for the RCT, who was recruited at the same time as the refuser. By replacing 1:2 refusers (as opposed to 1:1), this retains the balance in the two intervention arms. We will also analyze the characteristics of the refusers qualitatively, to identify whether they are different from the individuals who agreed to participate in the intervention.

Intention to treat analysis will be used when data are missing. However, every effort will be made to minimize missing data. For instance when subjects are unable to be contacted at the agreed time for any one data collection point, we will note their data as missing; however, reasons for loss to follow-up will be sought by making further contact with the subject. If possible, replacement telephone interviews will be conducted within days of the missed appointment, to provide the missing information. When subjects provide some data during the telephone interview, but not all, reasons for missing information will be recorded, to determine the appropriateness of using ‘carry-forward last known value’.

### Analysis of data from Studies 1 and 2

The three study cohorts will be assessed at time of allocation into a study arm (one-month after recruitment) for homogeneity in key socio-demographic characteristics and immutable factors (for example, gender, age, schooling, postcode, diagnosis) and all outcome measures (including the percentage of ‘frequent flyers’ in each arm). We previously reported this as one or more ED contacts per month over the preceding 12 months [33,34].

Change over time in each outcome measure will be calculated using repeated measures analysis of variance (ANOVA) models, with study contact point, cohort (1, 2, or 3) and key demographic features as independent variables. Partial Least Squares (PLS) models using pathway analysis will be applied to the multiple outcome, immutable and mutable independent variables at each time point, to determine different spatial arrangements and the significant factors which impact on data clusters.

### Hypotheses

**Figure 2:**
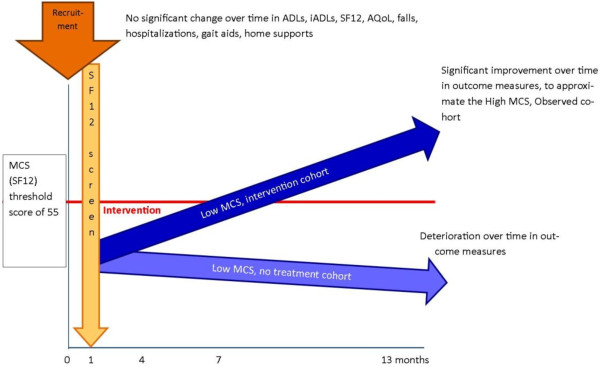
Hypothesized FD outcomes for the study groups.

As shown in Figure 2, our hypothese are:

1. The observed cohort will not demonstrate FD over the following 12 months in any FD measure.

2. The low SF12-MCS, no treatment cohort will demonstrate significant FD in any one or more outcome measures, taken over the next three months, when compared to their baseline recruitment status.

3. The low SF12-MCS, intervention cohort will show no FD (comparable findings to the observed cohort) over the next 12 months.

4. Additionally, those individuals in the low SF12-MCS, no treatment cohort who demonstrate significant FD at three months follow-up, and who access ‘rescue’ intervention (see Section on Ethics), will show an attenuated rate of FD over the following twelve months, compared with the rate they exhibited in the first three months.

### Timelines

**Figure 3:**
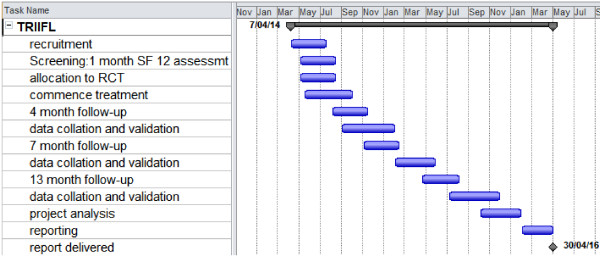
Timelines and tasks.

Based on our pilot work understanding of data collection issues, we have confidence that this project can be conducted successfully over 24 months (Figure 3).

#### Outcomes and significance

Given the high and increasing costs of tertiary healthcare, it is of increasing importance to communities and healthcare budgets, that incipient FD is detected early and effectively addressed. We propose that early sensitive screening coupled with low cost, individually-targeted interventions will save huge avoidable downstream health costs and assist older people to live safely and independently for longer at home.

The TRIIFL study aims to demonstrate that a simple early screening phone call to older individuals after discharge from ED to assess their physical and mental quality of life will identify those who are not coping (on the cusp of FD). By referring them then, to cost-efficient personalized community programs, significant improvements can be made by arresting or slowing FD. This research could shift Australian approaches to FD from a reactive acute care mentality to a proactive collaborative preventive approach. The benefits, at a time of diminishing public sector resources and increasing pressure on aged care and acute services facing rising complexity and demand, are considerable.

## Abbreviations

ADL: activities of daily living; AQoL_4D: Assessment of Quality of Life; ED: emergency department; ECH: elderly citizens homes (the intervention provider); FD: functional decline; FROP-COM: falls risk for older people- community settings; GP: general practitioner; HARP: Hospitals Admission Risk Profile; iADL: instrumental activities of daily living; MMSE: Mini Mental State Examination; QALYs: Quality adjusted life years; RCT: randomized controlled trial; SF12: Short Form-12 Health Survey; SF12-MCS: mental component scores of the SF12.

## Competing interests

The authors declare that they have no competing interests.

## Authors’ contributions

KG was responsible for overall trial design and reporting, and leading the pilot projects which preceded this protocol development. JL was responsible for input into the trial design in terms of inclusion of appropriate literature, and details on recruitment, sample size calculation and designing the intervention. KB was responsible for the management of the pilot functional decline project which preceded this protocol development, the literature review underpinning this protocol, and for input into the sample size calculation criteria. SK was responsible for input into the trial design and input into the projects which preceded this protocol development. AC was responsible for literature interpretation and input into the trial design. KP was responsible for providing expert input into the aged care system, in particular the intervention components and surrounding literature. All authors read and approved the final manuscript.
